# Pathogenicity of Virulent Species of Group C Streptococci in Human

**DOI:** 10.1155/2017/9509604

**Published:** 2017-06-12

**Authors:** Marta Kłos, Jadwiga Wójkowska-Mach

**Affiliations:** Department of Microbiology, Jagiellonian University, Czysta 18, 31-121 Kraków, Poland

## Abstract

Group C streptococci (GCS) are livestock pathogens and they often cause zoonotic diseases in humans. They are Gram-positive, in mostly *β*-hemolytic and facultative anaerobes. Because of their close evolutionary kinship with group A streptococci (GAS), GCS share many common virulence factors with GAS and cause a similar range of diseases. Due to the exchange of genetic material with GAS, GCS belong to bacteria that are difficult to be distinguished from group A streptococci; GCS are often treated in microbiological diagnostics as contamination of the culture. This report focuses mainly on the pathogenicity of virulent species of GCS and their association with human diseases. The condition that is most frequently quoted is pharyngitis. In this paper, the virulence factors have also been mentioned and an interesting link has been made between GCS and the pathogenesis of rheumatic diseases among the native people of India and Aboriginal populations.

## 1. Introduction

Group C streptococci, according to Lancefield's work of 1933, belong to Gram-positive bacteria, catalase-negative and facultative anaerobes [1–4]. The division of streptococci into groups was made by Lancefield; the research concerned 106 strains of* Streptococcus haemolyticus* isolated from many sources (humans, animals, and dairy products) and was based on the carbohydrate C expressed in the wall of bacteria [4]. Using sera, agglutination reactions, and reactions of precipitation Lancefield classified the strains into respective groups [4]. Based on the results of the anti-C precipitin test, it was stated that the strains of group C came from various sources other than human and numbered 49 strains. Those strains of group C can hardly, or impossibly, be distinguished from human hemolytic streptococci [4].

GCS identification can be difficult due to their close kinship with group A streptococci—they have a common evolutionary origin, and they are well-known for their exchange of genetic material, share many of their virulence genes, and cause similar diseases [5]. The study of the epidemiology of human GCS infections has recently been made possible mainly owing to the development of modern methods of molecular diagnosis. GCS are mainly livestock commensals or pathogens, although they can also be the part of the human flora of the nose, throat, intestines, or skin [3, 6]. The main origin of virulent species of GCS that cause human diseases is animals, unpasteurized milk, and unpasteurized dairy products [2, 6–10]. Infections caused by streptococci can be invasive or noninvasive, and they cannot be distinguished merely on the basis of their clinical presentation [3].

Astete reports that* Streptococcus equi* is regarded as the immediate antecedent of group C streptococci and is also the cause of almost 2% of all infections caused by GCS [1].  Table 1 depicts the species currently belonging to GCS (Table 1). According to Facklam GCS contains four species:* S. dysgalactiae* subs.* dysgalactiae*,* S. dysgalactiae* subs.* equisimilis*,* S. equi* subs.* equi*,* S. equi* subs.* zooepidemicus*, and* S. constellatus* subs.* pharyngis* [11]. The streptococcus of group C, which most often causes infection in humans, is* Streptococcus dysgalactiae* subs.* equisimilis* (SDSE) [10, 12]; it may cause skin infections (cellulitis) and recurrent blood infections [13]. According to Clarke the GCS species, not mentioned before, most often isolated from the human throat is* S. milleri* [8], also known as* S. anginosus* group [11].

## 2. Identification

In contemporary laboratories the identification according to the carbohydrate group GCS strains may be conducted by means of latex agglutination [5, 13]. Aside from the classification according to the carbohydrate group, there are many other identification methods, which will allow us to discriminate species within GCS and to discriminate GCS from other streptococci. Some selected phenotypic features of the GCS species are shown in Table 1 (Table 1).

Phenotypes of group C streptococci and group G streptococci (GGS) form a large colony, which makes them different from the phenotypes belonging to the* S. anginosus* group, which form a small colony, irrespective of the carbohydrate group [7]. The aforementioned* S. anginosus* group (called* S. milleri* by some authors) contains three species:* S. anginosus*,* S. constellatus*, and* S. intermedius* [7, 8, 11, 15]. The species that belong to the* S. anginosus* group contain in their cellular wall the carbohydrate groups A, C, F, and G or they contain none [11]. GCS and* S. anginosus* group can be identified on the basis of *β*-hemolytic activity [5]. All the species of the* S. anginosus* group and almost all the phenotypes of GCS cause beta-hemolysis except* S. dysgalactiae* subs.* dysgalactiae* [9, 11]. In order to discriminate GCS or GGS from GAS the PYR test is applied (the L-pyroglutamyl amino-peptidase test) [5]. The PYR test shows a positive result only in the case of GAS; both in the case of GCS and in the* S. anginosus* group no positive reaction had been found [5, 11].

In Angeletti's work we find two reliable optochin sensitivity and bile solubility tests, the tests mentioned also in Facklam's observation [11, 16]. Facklam shows that virulent streptococci, including those from group C, manifest a negative reaction in optochin sensitivity and bile solubility tests [11]. Aside from the aforementioned methods, Clarke et al. report that GCS can also be differentiated from other species through their capacity for sorbitol and trehalose fermentation ([8], Table 1). Among the phenotypes belonging to GCS, only S.* equi* subs.* zooepidemicus* can ferment sorbitol, whereas S.* dysgalactiae* subs.* dysgalactiae* manifests variable reactions [2, 11]. Almost all the phenotypes of GCS, except S.* equi* subs.* equi* and S.* equi* subs.* zooepidemicus*, which manifests a variable response, ferment trehalose [11]. It is interesting to note that none out of the 49 strains attributed in 1933 by Lancefield to group C fermented trehalose [4].

One can include within the molecular techniques of streptococci identification the* emm* typing, MALDI-TOF MS method (Matrix-Assisted Laser Desorption/Ionization Time-of-Flight Mass Spectrometry), and the sequencing of selected genes. The examination of the presence of* emm* and* emm*-like genes, called* emm* typing, allows us to determine the M protein serotypes [17]. In Dinkla's research the* emm* types were identified by means of PCR analysis, with the use of starters recommended by the Center of Disease Control [18]. In the case of GAS the* emm* gene sequence technique is combined with T typing, a fact that is not possible in GCS or GGS, for GCS/GGS contain no T proteins [17].

MALDI-TOF MS identification is based on the protein composition of cell bacteria, especially ribosomal proteins. In Angeletti's tests 158 strains have been isolated, all of them belonging to the viridans group streptococci (VGS), and examined in the MALDI-TOF MS; then genes* tuf*,* sodA, *and* rpoB* were amplified and sequenced [16]. It has been stated that in Angeletti MALDI-TOF MS sensitivity obtained 100% in comparison with the phenotypical tests [16]. To identify* S. dysgalactiae* subs.* equisimilis* strains Ciszewski presents the RISA method (analysis of ribosomal intergenic region) [19]. In the RISA method, in the course of PCR reaction, one uses* S. dysgalactiae* species-specific primers and SDSE subspecies-specific primers [19].

An alternative for the aforementioned molecular identification can be the sequencing of the 16S rRNA, in combination with* gyrB* gene sequencing generally regarded as a golden standard [16, 19, 20]. Zhou's test confirms that by having examined 181 isolates the similarities with the sequences in GenBank reached over 99% and 96% [20]. According to Zhou et al., however, the sequencing of the* gyrB* gene can be more practical and more precise in identifying VGS strains than other methods [20].

The basis of species identification appears to be the phenotypical tests used by many researchers; they confirm the fact that they belong to a given species and that the findings of MALDI-TOF MS are correct. Molecular techniques allow us also to detect or confirm the presence of some features which cannot be examined by means of the phenotypical methods of biochemical tests. These two groups of methods are inseparably linked, and in order to precisely examine the group C streptococci one should combine the phenotypical methods with molecular techniques.

New methods of identification based on protein typing or genome typing are barely available in Polish laboratories; therefore latex tests prevail. Probably, such a situation is also typical of other East and Central European countries, or in the developing countries. Therefore literature contains mainly the findings from the highly developed countries.

## 3. Virulence Factors

The virulence factor in some species of GCS most frequently mentioned is the M protein, the protein that also belongs to group A streptococci [2, 5, 7, 10, 12, 14, 21, 22]. This antiphagocytic, fibrillar, and surface-exposed M protein [14, 15] is encoded by the* emm* gene [12]. Bisno's tests have shown that M protein can mainly be found in* S. dysgalactiae* subs.* equisimilis* isolates isolated from patients suffering from acute pharyngitis [15]. There are over 80 well-known M protein serotypes of group A streptococci [15]. In comparative research between M proteins of GAS and M proteins of GCS isolates SDSE was examined from people and it was found that the structures of M proteins of GAS were highly homological to M proteins of GCS in the C repeat region but the N terminus was unique [15].

It seems that M protein plays an important role in rheumatic fever (RF) [5, 15]. McDonald and Bramhachari have noticed that GCS isolates more often than GAS colonize the throats of Aboriginal population suffering from RF incidence and rheumatic heart disease (RHD) [5, 21]. Dinkla's work seems to confirm that M and M-like proteins of group C streptococci take part in the pathogenesis of rheumatic fever in some geographic regions [18]. All of the 70 isolates* S. dysgalactiae* subs.* equisimilis* belonged to GCS/GGS; 27 of them had the ability to bind collagen IV and these were subjected to further analysis [18]. Out of 27 SDSE strains containing the* emm* gene, coding M proteins, 4 had the* fog* gene coding M-like fibrinogen binding protein of GGS (FOG) [18]. These findings are confirmed in Haidan's and Rantala's reports where GCS/GGS isolates were found, and the isolates that have the fibronectin-binding protein property can aggregate human platelets and can toxically affect the endothelial cells [13, 23]. Dinkla's research shows that 27 SDSE strains owe their ability to bind collagen IV to the hyaluronic acid capsule [18]. Furthermore McDonald and Nataneli describe the capacities of GCS for cross reaction with cardiac epitopes and their superantigenic features [21, 22].

Aside from M protein, some species of GCS (Table 2) can produce a surface-exposed protein (Szp), streptokinase, streptolysin O or streptolysin S [2, 10], and hemolysins [7]. Surface-exposed protein is produced by* S. equi* subs.* zooepidemicus* [11]. Chandnani indicates the SDSE strain to produce streptokinase and streptolysin O and shows theories about the pathogenesis of rheumatic fever [2]. There is a direct cytotoxic influence of streptolysin O on the cardiac cells and other organs or there are antigenic determinants between the components of an organism and some specific tissues, such as the brain or heart, that is a result of immunologic cross reactivity [2]. Following a comparative analysis of the GAS genome with the SDSE genome, Silva and Watanabe confirm that they contain related virulence factors [10, 14].

Besides the aforementioned virulence factors SDSE isolates have C5a peptidase, glyceraldehyde 3-phosphate dehydrogenase, and hyaluronidase [10]. There are also several important GAS virulence factors, which are not found in the SDSE genome, for example, cysteine protease, and a superantigen homolog [14]. SDSE enzymes, matrix metalloproteinases, and plasminogen binding GAPDH enzyme contribute to dissemination in the body and proliferation. An analysis of the genome of SDSE 167 strain has shown that it excretes lyases decomposing carbohydrates of the host, while at the same time this genome metabolizes carbohydrates in the Entner-Doudoroff pathway; this region contains the enzyme metabolizing polysaccharides, which may contribute to a higher virulence of SDSE 167 strain [14]. Watanabe et al. claim that SDSE 167 strain is the most virulent human strain [14].

## 4. Pathogenicity

Among the diseases caused by the species of GCS the dominating ones are pharyngitis, meningitis, and endocarditis (Table 2). According to Danish data (1999–2002), GCS infections take the last place among streptococcal infections, but they constitute as many as 6% of all streptococcal infections [3]. This has also been confirmed by the Norwegian observations of 1999–2013, where out of 512 invasive infections GCS was the cause of 24 cases (4,7%) [12]. In both reports, the risk of infection increases with the patient's age [3, 12]. Among other factors that predispose one to infections are diabetes, tumors, cardiac conditions, and alcohol abuse [3], whereas Rantala adds immunosuppressive therapy, multiple morbidities, and skin lesions as potential predisposing factors [13].

The most frequent clinical form of invasive virulent species of GCS infections are infections of bones, joints, and blood infections, and the noninvasive infections of the upper respiratory tracts [12]. The mortality rate during the first 30 days of the disease, in the Danish research, equaled 21%, whereas in the Norwegian research it equaled 9%, and it increased with age and with respect to the kind of disease [3, 12]. Ekelund et al. reports that the highest rate concerned patients with GAS (23%) and GCS infections (21%) [3]; in Oppegaard et al. the mortality rate in GAS infections was 13% [12]. Both Ekelund et al. and Oppegaard et al. list similar diseases: skin infections (erysipelas, cellulitis), necrotizing fasciitis, and toxic shock syndrome [3, 12].

Rantala presents two cases of elderly patients with recurring bacteremia caused by the most frequent GCS isolates, SDSE, and a broad range of diseases caused by the following subspecies: pharyngitis, tonsillitis, skin infection, soft tissue infection, wound infections, erysipelas, cellulitis, necrotizing fasciitis, streptococcal toxic shock-like syndrome, pneumonia, septic arthritis, osteomyelitis, meningitis, endocarditis, and bacteremia [13].

The findings of the British Health Protection Agency and Watanabe are alarming. They have shown a 100% increase of bacteremia between 2006 and 2012 and an increase of SDSE infections in Asia, Europe, and the USA [12, 14]. Of particular interest are the reports about the relations between GCS with the pathogenesis of rheumatic fever in Australia and India [5, 21, 23] and first documented zoonotic diseases [6, 8, 9, 22, 24].

As has already been mentioned, though RF pathogenesis is not clear only in the upper respiratory tract infection, GAS may directly lead to rheumatic fever. Haidan's studies have shown that in Australia higher indicators of isolation of GCS have been found as well as the highest rate of RF-dependent morbidity in the world [23], a fact which, as researchers say, may be related to high RF and RHD morbidity among Aboriginal communities of Australia [5, 21].

Nataneli et al. have described an American case of poststreptococcal syndrome uveitis (PSU), which is the first documented case of complications following pharyngitis and caused by GCS infection [22]. The patient was a 24-year-old man with HLA-B27 positive genotype (this genotype predisposing an individual also to poststreptococcal reactive arthritis). Morsch, however, describes the case of a 72-year-old man, suffering from tophaceous gout, in whom olecranon bursitis developed as the result of a GCS infection [24]. The author notes that until now circa 15 cases of septic arthritis (GCS) have been documented, but none have been related to bursitis [24].

In publications one can find some data concerning zoonotic diseases. The first report describes the case of a 13-year-old boy who lived on a horse farm; he became ill with meningitis caused by* S. equi* subs.* equi* [6]. The second case is of a 13-year-old girl suffering from meningitis caused by* S. equi* subs.* zooepidemicus* [9]. A day before her illness she took part in horse races [9]. A description of GCS-dependent meningitis with cavernous sinus in an 18-year-old man is worth mentioning [8]. Virulent species of GCS can also cause, among other things, impetigo, epiglottitis, and polyarthritis [5, 6, 21].

## 5. Conclusions

1. Virulent species of group C streptococci, which cause infection in people, are most often animals' pathogens and belong to bacteria that are difficult to be identified. Due to the common origin of GCS and GAS the method of identification is to combine phenotypical and molecular techniques.
2. The study of the epidemiology of human GCS infections is possible owing to the application of modern NGS (Next Generation Sequencing) methods in routine investigations.
3. Some species of GCS, chiefly SDSE, have many virulence factors found in GAS like M protein, streptokinase, and streptolysins, but there are some that are missing like cysteine protease. There are assumptions that virulent species of GCS can be related to the pathogenesis of rheumatic fever and rheumatic heart disease, especially among Aboriginal population of Australia.
4. The main origins of virulent species of GCS are unpasteurized dairy products, animals, and humans.
5. The most frequent diseases are pharyngitis and meningitis. The most frequent GCS isolates from human infection is SDSE.
6. The predisposing factors are, among other things, age and chronic diseases. The range of GCS-dependent diseases is broad, including infections related to the respiratory system, skin infections and soft tissue infections, necrotizing fasciitis, streptococcal toxic shock syndrome, septic arthritis, osteomyelitis, endocarditis, and bacteremia.

## Figures and Tables

**Table 1:** Phenotypic characteristics of group C streptococci.

Species	Lancefield group	*β*-hemolysis	PYR test	Sbl^a^	Tre^b^	Origin	References
*S. dysgalactiae* subs. *dysgalactiae*	C	−	−	v	+	Animals	[11]
*S. dysgalactiae* subs. *equisimilis*	A, C, G	+	−	−	+	Human, animals	[2, 5, 7, 10–12, 14]
*S. equi* subs. *equi*	C	+	−	−	−	Animals	[11]
*S. equi* subs. *zooepidemicus*	C	+	−	+	v	Human, animals, unpasteurized dairy products	[2, 11]
*S. constellatus* subs. *pharyngis*	C	+	−	−	+	Human	[11]

^a^Sorbitol fermentation.

^b^Trehalose fermentation.

−, negative reaction; +, positive reaction; v, variable reaction.

**Table 2:** Virulence factors and morbidity against human of group C streptococci.

Species	Virulence factors	Disease	Origin	References
*S. equi *subs. *equi*	Protein	Septic arthritis, meningitis	Animals, human	[1, 6, 11]
*S. equi* subs. *zooepidemicus*	Surface-exposed protein (Szp)	Meningitis, bacteremia, endocarditis	Unpasteurized milk; animals	[6, 9, 11]
*S. equi* subs. *zooepidemicus* *S. dysgalactiae* subs. *equisimilis*	Surface-exposed protein (Szp) *emm* gene homologs, streptokinase, streptolysin O and S, M protein, hemolysin, C5a peptidase, glyceraldehyde 3-phosphate dehydrogenase (GAPDH), hyaluronidase	Pharyngitis, pneumonia, endocarditis, meningitis, acute glomerulonephritis, skin and soft tissue infections, septic arthritis, osteomyelitis, endometritis, streptococcal toxic shock-like syndrome, necrotizing fasciitis, cellulitis, septic arthritis, bacteremia, RF^a^, RHD^b^	Unpasteurized dairy products, animals, human	[2, 5, 7, 9–11, 13]

^a^RF: rheumatic fever.

^b^RHD: rheumatic heart disease.
